# Theoretical Study of the Effect of Fibre Porosity on the Heat Conductivity of Reinforced Gypsum Composite Material

**DOI:** 10.3390/polym14193973

**Published:** 2022-09-23

**Authors:** A. Shalwan, Abdalrahman Alajmi, B. F. Yousif

**Affiliations:** 1 Department of Manufacturing Engineering Technology, Public Authority for Applied Education and Training, Kuwait City 13092, Kuwait; 2 School of Chemistry, Physics and Mechanical Engineering, Queensland University of Technology, Brisbane, QLD 4000, Australia; 3 Department of Power and Desalination Plants, Ministry of Electricity and Water, Kuwait City 12010, Kuwait; 4 Centre of Excellence in Engineered Fiber Composites (CEEFC), Faculty of Engineering and Surveying, University Southern Queensland, Toowoomba, QLD 4350, Australia

**Keywords:** porosity, heat conductivity, sisal fibre, glass fibre, gypsum, composite

## Abstract

In recent years, there has been an increasing demand for engineering materials that possess good mechanical and thermal properties and are cheap an d environmentally friendly. From an industrial and academic point of view, there is a need to study the heat conductivity of newly developed polymer composites and the influence of porosity on the insulation performance of polymer composites. Experimental and theoretical studies were conducted on mainly sisal/glass fibre gypsum composites with different fibre volumes (0, 20, 25, 30, and 35 wt.%). The outcomes from the theoretical model in ANSYS have shown that there is a high possibility to simulate the experimental work and high accuracy for reflecting the experimental findings. Moreover, the results show that natural fibre polymer composites with a high-volume fraction of natural fibres have higher insulation performance than synthetic polymer composites with the same volume fraction of synthetic fibres. Furthermore, the results suggest and support that the improved performance of natural fibre-based composites was due at least in part to the internal porosity of the fibres.

## 1. Introduction

This literature reviews the basic science of heat conductivity in fibre polymer composites and natural fibres. Composites are manufactured by combining raw materials in two phases: resin and a reinforcing material. The resin material can be either ceramic, metal, or polymer in which fibres are embedded to reinforce; in the case of polymers, the resins are normally thermosetting polymers. On the other hand, the reinforcing material can be fibrous or in any other form. It is normally synthesised material such as carbon, glass, or aramid fibres. Natural fibres based on wood or cellulose can also be incorporated into a biodegradable polymer matrix producing a new class of composites, termed green composites. The reinforcing fibres provide structural integrity and improved mechanical properties to the composite. Combining the two different phases produces a superior material called advanced fibre reinforced composite.

Among the synthetic composites, fiberglass composites hold the maximum market share because of their wide-scale applications. Advanced natural fibre reinforced composites are also investigated to make them competitive in manufacturing cost, performance, and long-term stability [1,2,3]. There is an increased interest in their use for various applications, such as in low-cost housing and other civil structures, where materials have lightweight yet good strength, and low environmental impact is required. There are many natural fibres such as sisal, banana, coir, and jute. That has the potential for reinforcing polymer matrix composites [4]. 

Researchers have investigated the amount of natural fibre needed to reinforce polymer mortar composites to improve their fracture resistance. However, natural fibre composites are not exposed to tough mechanical impacts such as synthetic fibre composites, which are, to a larger degree, used in high-tech engineering applications such as in the automotive and aerospace industries [5,6]. In the last decade, polymer composite materials have received a lot of interest, and their production has moved from laboratory research to industrial application. The main attraction of natural fibres over synthetic fibres is that they are abundantly available in nature, are renewable, have a low cost, and are recyclable [7,8,9,10,11].

### 1.1. Fibrous Reinforcement in Polymers

The primary objective of incorporating fibrous material into a brittle polymeric matrix is to improve its mechanical properties. Generally, fibre is defined as an elongated discrete piece or a bundle of threads composed of continuous filaments. Fibres which are thin and possess a longer surface-to-volume ratio usually adhere better to the polymer matrix. The arrangement of fibres in a polymer matrix can be conducted in two orientations, quasi-isotropic or orthotropic: in the quasi-isotropic orientation, all fibres are oriented in either single or multiple directions. In the orthotropic orientation, the fibres are oriented in multiple directions (but always at right angles to each other). Mechanical properties of the fibre reinforced composite also depend on the orientation. The type and orientation of the fibres depend upon the intended application [8,12]. The reinforced material may be required to have such features as excellent mechanical properties, lightweight, low-cost, and convenient availability. They should also have long-term stability and resistance to environmental variations. Compatibility with the polymer matrix, an integral material, and compatibility with the fabrication process. These properties are the key characteristics desired by the fibres synthetic, natural, and mineral. The synthetic and natural fibres are further elaborated on in the subsequent sections [13,14].

Synthetic fibres (also called man-made fibres) are made from various organic or inorganic materials. Examples of organic fibres include fibres made from ultra-high density polyethene and aramid (also commercially called Kevlar). Fibres made from carbon and glass are the most widely used inorganic fibres. Their synthesis can be either through mechanical extrusion or by any chemical method. Incorporating such fibres imparts improved mechanical properties to the composite such as high strength and stiffness, but they also add cost when compared to conventional engineering materials. They also pose environmental problems [12]. Compared to their different densities, high modulus carbon fibre has the highest specific modulus and tensile strength among all synthetic fibres. E glass fibre, which has a low and adequate modulus and tensile strength, is widely used in engineering applications due to its low cost.

Natural fibres are those found in nature and can be obtained from mineral, plant, or animal sources. The building blocks of plant fibres are mainly composite of cellulose microfibrils which are bound together by lignin and hemicellulose. The amount of cellulose depends on the type of fibre, and is usually 60 to 80% by weight. The percentage of lignin is around 5 to 20% by weight, and the moisture contents can range up to 20% by weight. Plant-based fibres such as cotton, jute, and flax have cellulose as the primary building block, and as a result, their fibres are highly polar. This polarity is caused by the hydroxyl groups present on the surface, and when such fibres are embedded in a polymer to form a composite material, these hydroxyl groups form hydrogen bonding with the matrix. Animal fibres such as wool, silk, and hair, on the other hand, consist of proteins. Mineral fibres are rarely used and investigated because of their carcinogenic nature [7,15,16].

### 1.2. Porosity in Natural Fibre-Based Composites

Porosity is commonly referred to as empty spaces or cavities that develop in a continuous material during processing or due to the air that becomes trapped during the processing of the material. In composites, such cavities are developed during different processing steps such as mixing or consolidation of fibres and the polymer resin. In the case of composites based on synthetic fibres, significant knowledge is present on how to control the porosity during the processing stage. In addition, the effect of this porosity on the mechanical and thermal properties is also well known [17,18,19]. In the case of those composites which are made from natural fibres, the knowledge about the control of the porosity and its effect on the different properties (mechanical and thermal) of the final composite is very limited. Considering the fact that the porosity can contribute to around 30% of the total volume fraction of the composite, it needs consideration for obtaining desired properties of natural fibres-based composites [17,20]. Some of the researchers have proposed a method of porosity determination by using mathematical models. By using a number of empirical parameters in their model, they predicted the porosity as a function of fibre weight fraction [17]. In addition, the model also enabled the calculation of matrix and natural fibre volume fractions. It has also been illustrated that the presence of cavities in the form of long capillaries also influences the composite properties. These capillaries strongly influence the permeability and heat transfer across the material. The development of a relationship between the porosity and thermal conductivity is usually not straightforward because it varies with the type of fibre and the polymer matrix [21].

### 1.3. Heat Conductivity of Polymers

It is well known that polymers have many applications in several industrial sectors. Many academic researchers have studied the electrical conductivity of polymers. Composites have a sensitive feature of electrical conductivity in polymer-based composites; most of the applications in electronics and electrical requires electronically conductive polymer composites, numerous trading devices such as bipolar plates and gas flow layers in Proton Exchange Membrane (PEM) fuel cells, conductive pastes comprising the conductive stuffing and polymer resins. Therefore, according to the increase in the mechanical stronger polymers need to have higher electrical conductivity. The best way to study the electrical conductivity is to simulate and model the behaviour of the polymer because of the effectiveness and the low cost that these methods provide. Moreover, investigation of the properties of the polymer matrix, it is reinforcement, and the interface between their naturals can be studied by this modelling. Several ways can be used to model the electrical conductivity in polymer-based composites. Investigators used numerical simulation [22,23,24,25], resistor modelling, image processing, and analytical and mathematical modelling [24]. There are several works that have been recently conducted to instigate the electrical conductivity of such materials. Despite that, there is a lack of knowledge about the heat conductivity of these materials [26]. In recent years, there has been ongoing research conducted to investigate the heat conductivity of polymers and their composites due to their vast applications. Tanaka and Ogata [27] reported that heat dissipation in composite polymers is essential for compact electrical devices but for different engineering applications. Thermal conductivity is the ability of a material to transfer heat. The method is according to the ASTM C518 procedures. According to research papers, natural fibres can decrease a composite’s thermal conductivity and therefore allow it to be used as an insulator in a building. Chikhi and Agoudjil [28] developed a new bio composites material as thermal insulation in buildings. The fibre is date palm fibres. From their experimental investigations, the thermal conductivity increases with the introduction of date palm fibre. The composite’s compressive and flexural strength can be improved by adding adequate fibre content. This kind of new bio composites is able to be used in buildings for thermal insulation.

According to Binici and Aksogan [29] found that the fibre-reinforced mud bricks had better thermal insulation and mechanical properties according to both ASTM and Turkish standards. The testing results showed higher compressive strength and heat conductivity than concrete brick. Korjenic and Petránek [30] used jute, flax, and hemp to develop new insulating materials for buildings. Their thermal conductivity test results revealed that natural fibre composites are likely to become a suitable alternative to commonly used boards. Panyakaew and Fotios [31] made a low-density thermal insulation board from coconut husk and bagasse. It is found that the bagasse insulation board has a low density of 350 kg/m^3^ and a thermal conductivity value from 0.046 to 0.068 W/mK, which is comparable to cellulose fibres and mineral wool. Actually, there are current trends that can be characterised as trends of increasing quality requirements for structures connected to growing awareness towards using materials with less environmental impact such as larch bark [32] and recycling residues of agricultural production [33,34].

### 1.4. Heat Conductivity of Natural Fibre Based Composites and Porosity

The composite polymers reinforced with natural fibres also offer good acoustic properties and thermal insulation. A number of research studies have determined the thermal conductivity of composites made by combining resins such as polypropylene, polyesters, and soya bean oil with natural fibres such as flax, cellulose, pulp, recycled paper, and chicken feathers [35,36,37]. These studies focused on the effect of different natural fibres on the thermal and mechanical properties of conventional polymer resins. In order to incorporate natural fibre-based composites in structural applications, recently, date palm fibres were used for making bio-composites. The experimental investigations revealed that the thermal conductivity of the composite increases after the addition of date palm fibres. In addition, the compressive and flexural properties were also improved with the addition of adequate fibre contents [38]. Similarly, sandwich beams made of natural composites have been studied for structural applications. A number of natural fibres such as flax, recycled paper, cellulose, pulp, and chicken feathers are added to a resin obtained from soya bean oil. It was found that the incorporation of natural fibres improved the mechanical properties of the sandwich beam, but they also increased the thermal and acoustic resistance properties [35]. Other studies have also reported thermally insulating materials such as boards made from coconut husk/bagasse and composites made from flax/kenaf/hemp/sisal reinforced polypropylene. Both these materials showed good mechanical properties and an increase in thermal insulation, which increased their suitability as structural materials [36,39,40].

### 1.5. Heat Conductivity of Natural Fibre-Based Composites

There are several methods that can be used theoretically and experimentally to assess the thermal conductivity of a material. The main purpose is to determine the coefficient of thermal conductivity, which is expressed as W/mK. By appling the following equation, the conductive heat transfer through any material can be calculated by the following equation:

(1) $$k=\frac{QL}{A\Delta T},$$

where

*k* = is thermal conductivity in W/m K;
*Q* = is the amount of heat transfer through the material in J/s or W;
*A* = is the area of the body in m^2^;
Δ*T* = is a difference in temperature in *K*°;
*L* = length of the body in m.

Thermal conductivity is defined as the quantum of heat transmitted through a unit thickness of the material. It is used to assess and compare how simple materials transfer heat. Although, there is an inverse relationship between the thermal conductivity and heat insulation properties. Many investigators have investigated and tested the thermal conductivity of diverse materials so as to take out optimum thermal energy that would save the use of the appropriate materials to deliver their intended use. Liu and Takagi [41] studied the size of the lumen (the hollow part of the fibre bundle) on the impact transverse thermal conductivity of unidirectional natural fibre composites. Through computer modelling, the unidirectional natural fibre composites were modelled in a two-dimensional SAPF. To evaluate the effective transverse thermal conductivity of this composite model thermal-electrical analogy technique needs to be thoughtful.

It was observed that the geometrical ratio *β* is the most important parameter for the dimensionless effective transverse thermal conductivity Kt+ of natural fibre composites. It is concluded that the most effective the transverse thermal conductivity of natural fibre composites as compared to conventional fibre composites are as follows:

(2) The thermal conductivity ratio β=Kf/Km

where *f* and *m* represent fibre and matrix, respectively.

Liu and Takagi [41] have tested the transverse thermal conductivity of unidirectional epoxy composites reinforced with abaca and bamboo fibres using. Resign transfer modelling (RTM) technique. by using them based on the impact of the microstructure of natural fibre. It has been observed from the results that the transverse thermal conductivity has an interdependent relationship with bamboo as the thermal conductivity increases with increasing bamboo fibre but decreases with increasing abaca fibres. On the other hand, it was found that the lumen structure plays a significant role instead of crystal structures and chemical components in the transverse thermal conductivity of unidirectional composites based on both microstructure and theoretical analysis. This information can be used to evaluate and design natural fibre reinforced composites with better thermal insulation properties. According to Ramanaiah, Ratna, and Prasad [42], it was discovered that the thermal conductivity of Typha angustifolia fibre reinforced polyester composites decreases with an increase in fibre content. It was observed that the number of thermal conductivities gained from tentative empirical models were optimum with the agreement that have been experimentally measured values. In part of that, the composites under the investigation had good insulating properties that would be suitable for real-life applications. For example, electronic packages, insulation boards, automobile parts, and for building construction.

An evaluation has been conducted by Binici and Aksogan [43] evaluated the impact of stalk sample size with epoxy/corn stalk particle ratio on the thermal conductivity of the composites and their mechanical properties. Mechanical properties have been compared with commercially available bio-based insulation materials. The investigation has defined that it is possible to use bio-composite materials with minimum heat transfer coefficients. The developed composites have thermal conductivity coefficients lower than 0.1 W/mK, meeting the requirement set by TS 805 EN 601 in order to qualify a material as a thermal insulator. The researcher was satisfied with the effectiveness of the sustainable filler of organic origin.

Wang and Qin [44] reported that the overall thermal property of the composites increased with the increase in interface thickness for carbon and glass fibres. However, it decreased for composites model thermal property of hemp fibre decreases. It is concluded that the rising interface thickness will increase the volume fraction of the fibre and interface region. At the same time, the volume fraction of the special element manufactures lower thermal conductivity of the composite because the thermal conductivity of both interface and fibre materials has a lower thermal conductivity than the matrix. This is an example of why a higher fibre volume fraction can uncertainly result in either lower or higher effective thermal conductivity of the composites.

## 2. Numerical Process Methodology

**Figure 1:**
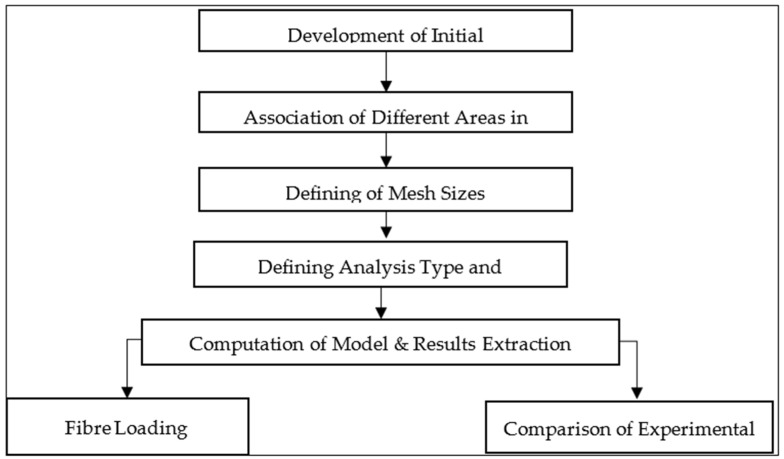
Flowchart of the development of a cylindrical model.

This study has been built based on an experimental work which has been achieved in the last period and published [45,46]. The Finite Element Method (FEM) was introduced in 1956. It is considered a powerful computational tool for the estimation of solutions to problems faced in real-life engineering. Physical phenomena occur in a continuum of matter ranging from solid to gas, subject to field variables. These field variables usually vary between points, and they possess a finite number of solutions within that domain. By using this tool, the problems are simplified by converting the whole domain into a finite number of pieces or elements. An approximate function could be associated with the unknown field variables. Modelling of thermal can be carried out by using FEM programs such as ANSYS [45]. In this research, ANSYS was used to analyse conductive heat transfer through fibre composite bodies. A number of practical heat transfer problems require the use of numerical methods, which allow problems to be solved quickly. The effect of changes in parameters can often be seen when a problem is modelled numerically. A numerical formulation can also be performed by using partial differential equations. These equations are replaced by discrete approximations, such as temperature fields that are approximated by the values at discrete points. As a result, a computational mesh is formed (in Cartesian or cylindrical coordinates), and the field is considered at consecutive time steps with a time increment Δ*t* [47]. This type of modelling of a cylindrical composite is explained in this chapter. In Figure 1, the flow of the numerical programmer of a cylindrical model is summarised.

### 2.1. Numerical Modelling of Cylindrical Coordinates

Numerical modelling of cylindrical coordinates and the heat transfer has been investigated and researched by many researchers [47,48,49]. Consider heat transfer across a cylinder whose radius is *r*, and the heat transfer is taking place in vertical and radial directions in rotational symmetry around the *z*-axis. Here, the temperature along with *r* in the *z*-direction at a specific time *t* is given by [48,49],

(3) T=T(r,z,t)

**Figure 2:**
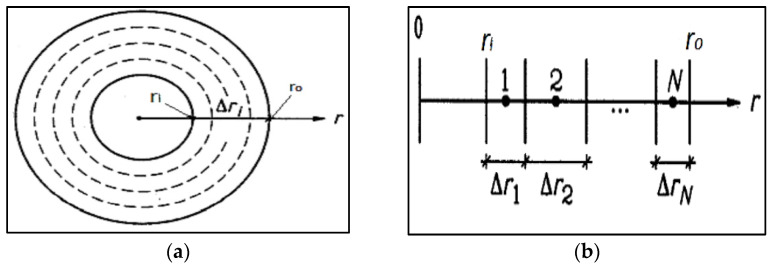
(**a**) Proposed cylinder for heat transfer study and (**b**) details of the meshes in the radial direction.

A radial and a vertical thermal process which is rotationally symmetric around the *z*-axis is considered for this modelling. Here, the interval along ‘*r*’ is divided into a mesh having cell widths (as shown in Figure 2a) as follows [48,49],

(4) Δri,   i=1,N

The inner boundary is at *r* = *r_I_*, the outer boundary at *r* = *r_o_*, and the midpoint is at *r* = *r_i_*. Here, *r_I_* may be at the *z*-axis and hence zero (as shown in Figure 2b).

Here we have [48,49],

(5) $$r_{1}=r_{I}+\frac{\Delta r_{1}}{2}$$

(6) $$r_{i}=r_{i-1}+\frac{\Delta r_{i-1}}{2}+\frac{\Delta r_{i}}{2}$$

In the above equation, ‘*i*’ ranges from 2 to *N*. If the widths of the individual cells are summed up, we obtain the total annulus width as follows [48,49],

(7) $$\sum_{i=1}^{N}\Delta r=r_{o}-r_{I},$$

**Figure 3:**
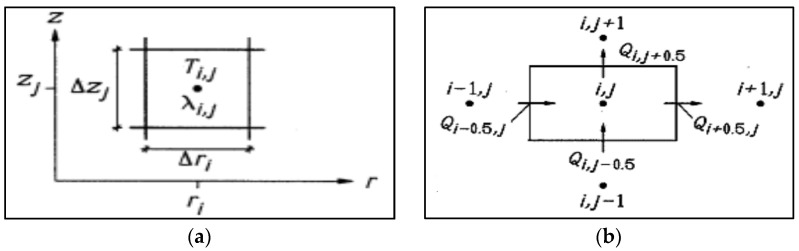
(**a**) A mesh element in the cylindrical coordinates and (**b**) illustration of heat flows (W) to and from the *Cell* (*i*, *j*).

The dimensions in the *z*-direction are shown in Figure 3a, and the cell widths in the *z*-direction are given as Δ*Z_j_*. At the middle point of *Cell* (*i*, *j*), the temperature at a time step of ‘*n*’ is given by the following relation [48,49],

(8) $$T_{i,j}=T\left(r_{i},z_{j},n\Delta t\right)$$

Here, the *Cell* (*i*, *j*) shown in the figure is an annular section of cylindrical shape and its dimensions are given as [48,49],

(9) $$r_{i}-\frac{\Delta r_{i}}{2}\leq r\leq r_{i}+\frac{r_{i}^{\prime }}{2}$$

(10) $$z_{j}-\frac{\Delta z_{j}}{2}\leq z\leq z_{j}+\frac{\Delta z_{j}}{2}$$

The volume of the cell can be given by using the dimensions of the cell by the following relationship [48,49],

(11) $$\Delta r_{i}\Delta z_{j}2\pi r_{i}=\left[\pi \left(r_{i}+\frac{\Delta r_{i}}{2}\right){}^{2}-\pi \left(r_{i}-\frac{r_{i}}{2}\right){}^{2}\right]\Delta z_{j},$$

The conductance (*K*) between two successive cells, i.e., *Cell* (*i*, *j*,) and *Cell* (*i* − 1,), could be given as [48,49],

(12) $$K_{i-0.5j}=\frac{\Delta z_{j}}{\ln\left(\frac{r_{i-0.5}}{r_{i-1}}\right)\left(\frac{1}{2\pi \lambda_{i-1,j}}\right)+\ln\left(\frac{r_{i}}{r_{i-0.5}}\right)\left(\frac{1}{2\pi \lambda_{i,j}}\right)+\left(\frac{r_{i-0.5}}{2\pi r_{i-0.5}}\right)},$$

The conductance in the above equation is in watts per kelvin (W·K). The thermal conductivity is given by the term for cells, respectively. In addition, *R*_*i*−0.5,*j*_ represent an optional thermal resistance present at the interface of two cells. The first term in the denominator gives the value of the thermal resistance per unit height of the *r*_*i*−1_ ≤ *r* ≤ *r*_*i*−0.5_ annulus. 

The heat conductance in the direction of the *z*-axis can be given as a product of the area of the cell perpendicular to the *z*-axis (2*πr_i_*Δ*r_i_*) and unidimensional conductance. The equation is given as follows [48,49],

(13) $$K_{i,j-0.5}=\frac{2\pi r_{i}\Delta r_{i}}{\left(\frac{0.5\Delta z_{j-1}}{\lambda_{i,j-1}}\right)+\left(\frac{0.5\Delta z_{j}}{\lambda_{i,j}}\right)+r_{i-0.5,j}}$$

The heat flows are shown in Figure 3b. They are given by the product of temperature difference and conductance. These flows are used for all ‘*i*’ and ‘*j*’ and are as follows [48,49],

(14) $$Q_{i-0.5,j}=K_{i-0.5,j}\left(T_{i-1,j}-T_{i,j}\right),$$

(15) $$Q_{i,j-0.5}=K_{i,j-0.5}\left(T_{i,j-1}-T_{i,j}\right),$$

In a time-step of Δ*t*, an increase or decrease in the temperature takes place, and as a result, *T_i_*, is used for the next time step. Hence, the heat balance equation takes the form as follows [48,49],

(16) $$\Delta r_{i}\Delta z_{j}2\pi r_{i}C_{i,j}\left(T_{i,j}-T_{i,j}\right)=\left[Q_{i-0.5,j}-Q_{i+0.5,j}+Q_{i,j-0.5}-Q_{i,j+0.5}\right]\Delta t,$$

The choice of a stable time-step for the *Cell* (*i*, *j*) is made by using the stability criteria given as [48,49],

(17) $$\Delta t<\left(\frac{\Delta r_{i}\Delta z_{j}2\pi r_{i}C_{i,j}}{\sum K}\right),$$

Here, $\sum K=K_{i-0.5,j}+K_{i+0.5,j}+K_{i,j-0.5}+K_{i,j+0.5}$.

The above criteria for numerical stability must be satisfied for the *Cell* (*i*, *j*), and the smallest time-step obtained is used to guarantee stability for all cells.

### 2.2. Numerical Theory behind ANSYS Modelling

The first law of thermodynamics states that thermal energy is conserved. Specialising this to a differential control volume, the equation of the following form can be given as (SAS, 2012) [48,49],

(18) $$Q=\nabla \cdot \left\{q\right\}+\left(\frac{\partial T}{\partial t}+\left\{v\right\}T\nabla T\right)\rho c,$$

In the above equation, the vector functions are further explained as [48,49],

(19) $$Vector\;operator=\nabla =\{\begin{array}{c} \frac{\partial }{\partial x}\\ \frac{\partial }{\partial y}\\ \frac{\partial }{\partial z} \end{array}\{\partial \;\partial x/\partial \;\partial y/\partial \;\partial z,$$

(20) $$Velocity\;vector\;for\;mass\;transport\;of\;heat=\left\{v\right\}=\{\begin{array}{c} Vx\\ Vy\\ Vz \end{array},$$

In the next step, the heat flux vector is related to the thermal gradients by using the Fourier’s law of heat conduction as follows [48,49],

(21) {q}=−[D] ∇T

Here, [*D*] is the conductivity matrix, which is given as follows [48,49],

(22) $$\left[D\right]=\left[\begin{array}{ccc} k_{xy} & 0 & 0\\ 0 & k_{yy} & 0\\ 0 & 0 & k_{zz} \end{array}\right]$$

In the above matrix, the *k_xx_*, *k_yy_*, and *k_zz_* are the conductivity elements in the *x*, *y*, and *z* dimensions, respectively. The equations could be combined to form a compound equation as follows [48,49],

(23) $$Q=\left(-[D]\nabla T\right)+\left(\frac{\partial T}{\partial t}+\frac{\partial T}{\partial t}+{\left\{v\right\}}^{T}\nabla T\right)\rho c,$$

(24) $$Q+\nabla \cdot \left(\left[D\right]\nabla T\right)=\left(\frac{\partial T}{\partial t}+{\left\{v\right\}}^{T}\nabla T\right)\rho c,$$

The above equation can be expanded to a more familiar form as follows [48,49],

(25) $$\left(\frac{\partial T}{\partial t}+v\frac{\partial T}{\partial x}+v\frac{\partial T}{\partial y}+v\frac{\partial T}{\partial z}\right)\rho c=Q+\frac{\partial }{\partial x}\left(K\frac{\partial T}{\partial x}\right)+\frac{\partial }{\partial y}\left(K\frac{\partial T}{\partial y}\right)+\frac{\partial }{\partial z}\left(K\frac{\partial T}{\partial z}\right),$$

Here, it has been assumed that all of the effects are in the global Cartesian system. There are three boundary conditions considered for this system, and it has been presumed that these conditions cover the entire element. These are given as follows,

- Specified temperatures acting over the surface S1, *T* = T*

The T* in above is the specified temperature.

**Figure 4:**
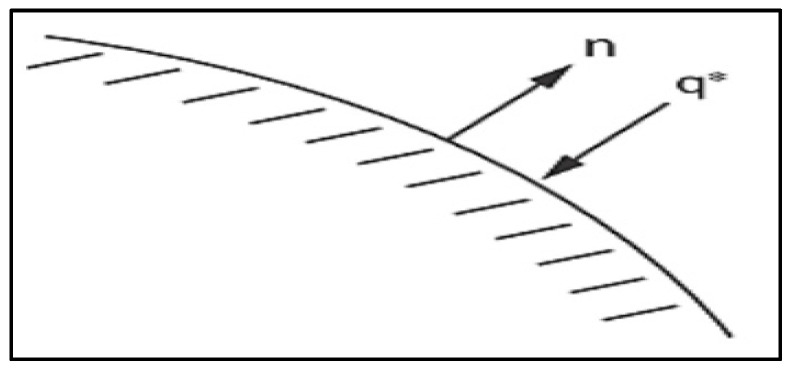
Specified convection surfaces.

- Specified heat flows acting over the surface S2 as shown in Figure 4 is given as [48,49],
  (26) −Q*={n}{q}T

Here,

{*n*} = Unit outward normal vector;
Q* = Specified heat flow.

## 3. Development of ANSYS Models

### 3.1. Formation of Preliminary Model Shape

**Figure 5:**
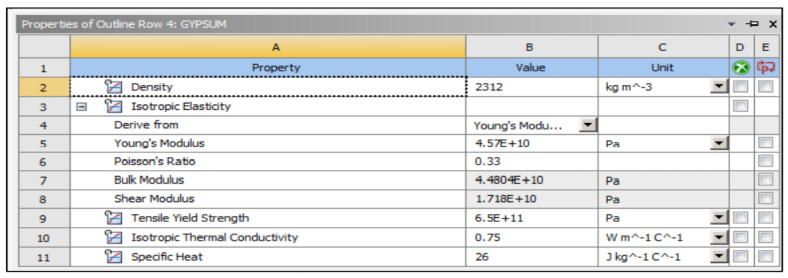
Mechanical properties of gypsum.

**Figure 6:**
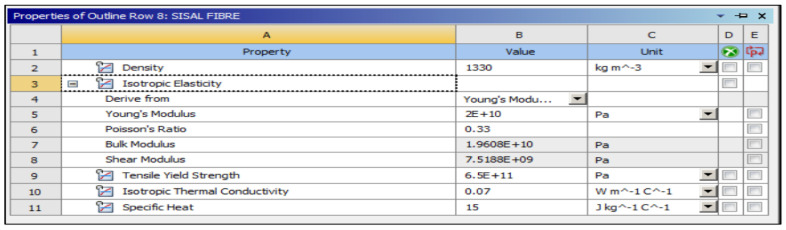
Mechanical properties of sisal fibre.

**Figure 7:**
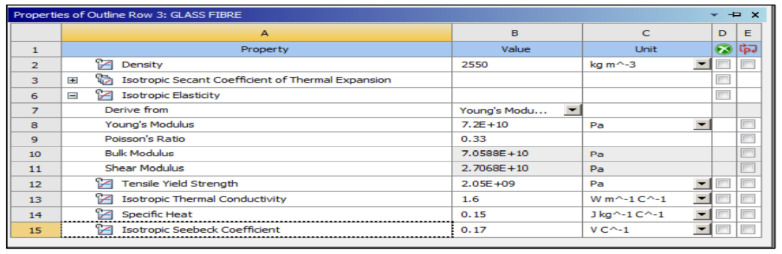
Mechanical properties of glass fibre.

**Figure 8:**
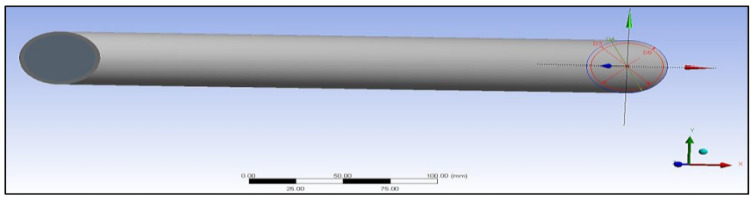
Initial model of gypsum cylinder without sisal fibres.

**Figure 9:**
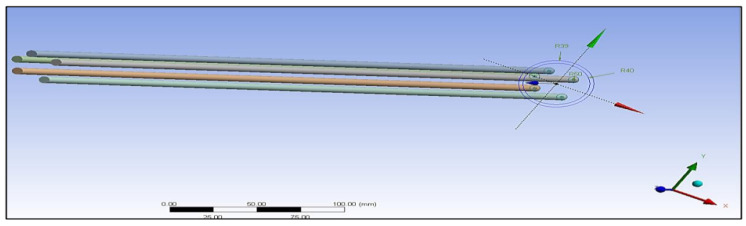
Modelling of the extruded sisal-glass fibres.

**Figure 10:**
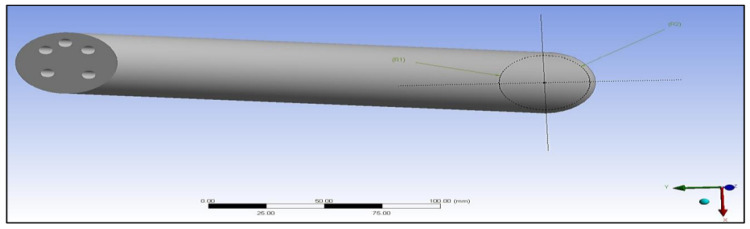
Simple model of a composite cylinder with surface points of fibres places.

The preliminary shapes of models and fibres were made by providing information about the elements and related material. The thermal properties of the three materials of composite cylinders are also given in Table 1. The geometries created are shown in Figure 5, Figure 6, Figure 7, Figure 8, Figure 9 and Figure 10, such as the initial model of a gypsum cylinder without sisal fibres and the modelling of the extruded sisal-glass fibres, and the simple model of a composite cylinder with surface points of fibres places.

All composites’ materials have been inserted in the engineering data of ANSYS, and all their mechanical properties will be shown from Figure 5, Figure 6, Figure 7, Figure 8, Figure 9 and Figure 10 properties that are most important and need to make sure that they have been entered correctly are the thermal properties such as heat conductivity and specific heat.

### 3.2. Generating the Mesh

**Figure 11:**
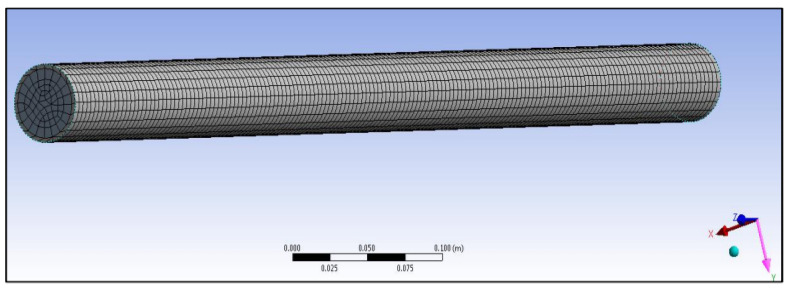
Meshed of the primary model of a gypsum cylinder without sisal fibres.

**Figure 12:**
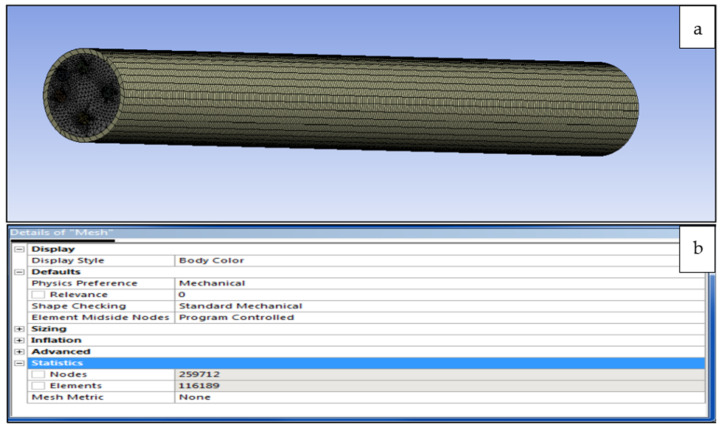
(**a**) Meshed model of composite cylinder incorporated with 20% sisal fibres and (**b**) is nodes and elements.

Meshing is the most important tool in computer simulation as it can make the results more accurate, irrespective of whether the simulation is structural, static, or thermal analysis. In addition, meshing is a very useful tool for generating a specific area in the model which needs a focused analysis. This makes the users more comfortable in using it in some specific cases. For example, in the project model, the temperature has been applied to one area in which the finest mesh is required as compared with the rest surfaces where a coarser mesh is sufficient. The different meshes created for each composite cylinder are shown in Figure 11 and Figure 12.

**Figure 13:**
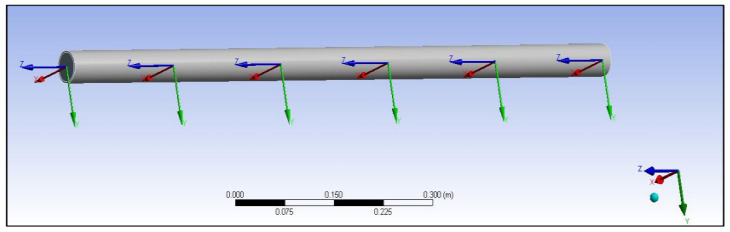
Orientation of the five parts of a composite cylinder in a coordinate system.

The meshed models were divided into five sections for analysing the heat flow across the composite cylinders. The total length of the cylinder was taken as 10 cm, and each section’s length into 2 cm. The temperature of the beginning of the cylinder was taken as 120 °C, and this model is shown in Figure 13 as follows. The orientation of the five parts in the coordinate system is also given along with in Figure 13.

### 3.3. Boundary Condition

**Figure 14:**
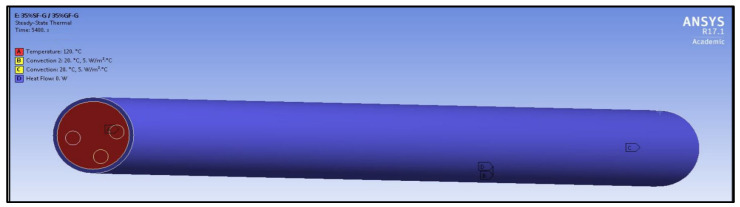
Boundary conditions of composite cylinder.

The boundary conditions are given in Figure 14; it can be seen that 120 °C has been applied on one side and on the end of the other side, and convection of 20 °C have also been applied to the covered insulated material, which is shown in the blue colour of zero heat flow and has convection of 20 °C.

### 3.4. Fibre/Polymer Modelling in ANSYS

**Figure 15:**
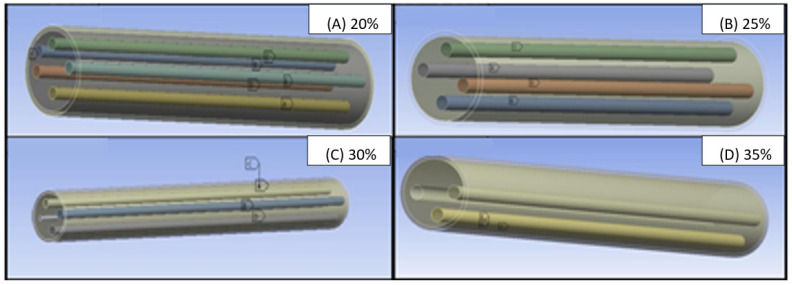
Model of composite cylinder incorporated with 20%, 25%, 30%, and 35% sisal fibres.

The model was then solved, and the solution of the simultaneous equations was obtained by the software to give images of the models in Figure 15. Moreover, the dimensions of the model cylinders are also given in Table 2.

## 4. Results and Discussion

### 4.1. Introduction

**Figure 16:**
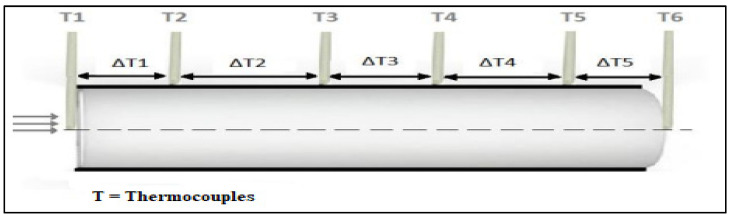
Temperature reading along with the cylinder [50].

The numerical results of the thermal characteristics of different fibre/matrix composites are presented in this section. The numerical test was evaluated on mainly sisal fibre and glass fibre which they combined with gypsum, as shown in Table 3. Prolonged heat exposure was conducted at the beginning of the cylinder with a duration of 90 min, and the measurements were taken at these points T1, T2, T3, T4, T5, and T6, as shown in Figure 16. Fibre volume fractions of the thermal conductivity of the composite have taken place. The comparison of the experimental and the numerical results is also evaluated.

### 4.2. Sample of ANSYS Results

#### 4.2.1. Gypsum with 20% of Glass-Fibre

**Figure 17:**
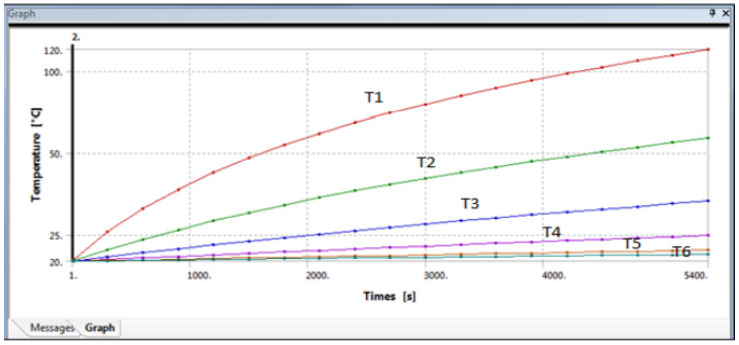
Variation of temperatures along the composite cylinder lengths with time.

**Figure 18:**
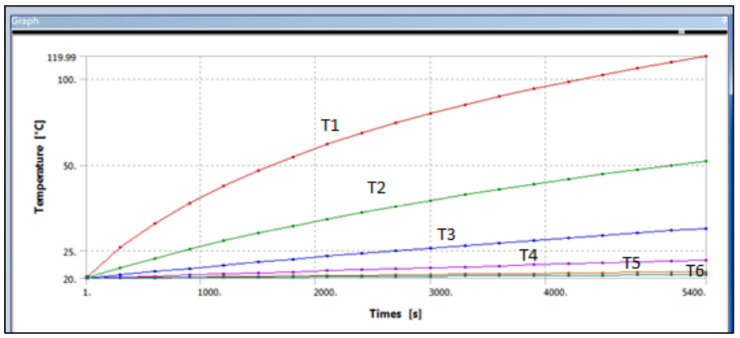
Variation of temperature along the composite cylinder lengths with time.

The results for gypsum with 20% glass fibre are presented in Figure 17 and Figure 18, that shows the temperatures at different points across the cross-sectional area of the composite cylinder. As shown previously in Figure 16, the cylinder was divided into multiple sections to assess the temperature distribution along the cylinder length. The temperature decreases from the heated end to the unheated end. Figure 17, for example, shows that the temperature varies from 56.5 °C to 21 °C for one observation. The graph in Figure 18 provides similar information about the changes in temperatures along with the timeframe. 

Figure 18 shows the different temperatures along with the glass fibre gypsum cylinder, which has taken from the ANSYS graph, and it can be seen that the first curve shows the highest temperature of around 120 °C and that refer to T1, the second curve of the following curve is showing T2 which is about 56.536 °C till the last curve which T6 which is about 21.127 °C. All the temperature differences in the simulated model were tabled in Table 4.

The error was calculated using

(27) $$\%Error=\frac{\left|EXP-NUM\right|}{EXP}*100$$

Table 4 shows all data from the experimental and numerical results. On the numerical side, it can be seen that with the increment of volume fraction of glass fibre at ΔT1 is slightly increasing until is an end to 25% it was dropping down. Glass fibre shows decreasing of heat transfer with compared to pure gypsum. The decrement of the insulation performance of pure gypsum is −7.1, −7.5, −1.2, and −6.6% for all volume fractions of 20, 25, 30, and 35%, respectively. This shows that the highest gap between T1 and T2 is the glass fibre, with a volume fraction of 25%. In addition, this results in making glass fibre more conducive to heat than pure gypsum, which means that glass fibre is better insulation performance compared to pure gypsum. However, that does not show an agreement with the experimental results as it showed that the highest gap in temperatures between T1 and T2 is the glass fibre with 35%, and that is obviously because of the mistake that might be involved while making the model of both 30% and 35% volume fraction. However, it did show agreement with both 20% and 25% of glass fibre. Some studies have been conducted by Cao and Liu [51] on thermal insulation features of glass fibre used for board building. It was defined that it is hard to be able to suddenly explain the porous that is inside a structure due to the complexity of the composites. It is well known that the heat conductivity drops with raising porosity of the near-linear rate. In the following Table 5, all the error percentage between the experimental and numerical results has been calculated using Equation (20).

#### 4.2.2. Gypsum with 20% of Sisal-Fibre

**Figure 19:**
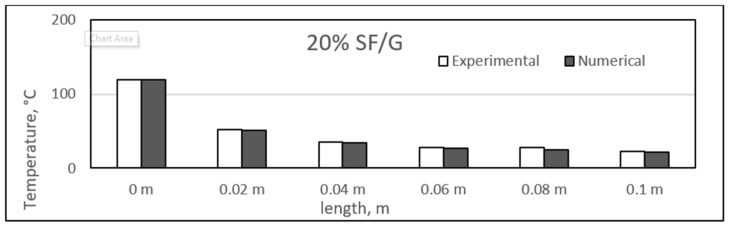
Comparison between experimental and numerical results of 20% sisal/gypsum.

Similarly, the ANSYS results for sisal fibre reinforced gypsum composite were exported from the software and are shown in Figure 18 and Figure 19. These images are of 20% sisal fibre volume fraction of the composite. The images ranging from Temperature 2 to Temperature 6 show the temperatures at different measurement positions across the cross-section of the composite cylinder. As compared to the glass fibres, we have a considerable difference in temperature for the sisal fibres at the first observation point. The thermal conductivity of sisal fibre composite was much less than glass fibre, with a temperature difference of 5 °C. Similarly, at the last observation point, the temperature forces in this sisal fibre composite are lower as compared to the glass fibres composite. The temperature varied from 51 to 20.5 °C across the length of the composite cylinder.

Figure 18 shows the different temperatures with respect to the total length of the cylinder with the sisal fibre-gypsum cylinder, which has been taken from the ANSYS graph, and it can be seen that the first curve showed the highest temperature of around 120 °C and that refer to T1. Secondly, the next curve shows T2, which is about 51.487 °C, till the last curve, which is T6, shows about 20.534 °C. All the temperature differences of the simulated model are tabled in the following Table 6.

From Table 6 and Table 7, temperature differences across sisal fibre-gypsum composites of both experimental and numerical, which obtained both experimental and numerical results by discussing the numerical results, it can be observed again that ΔT1 shows a slightly similar reading. The first temperature differences show increasing with respect to adding sisal fibre, from the vol.% of 35% shows the highest temperature differences from ΔT1 and ΔT2 indicates that the heat transfer between these two points is in its highest gap in temperature. This illustrates that with the addition of sisal fibre the thermal transfer decreases, which makes sisal fibre a better heat insulator performer. The increment in heat insulation feature is 3, 3.4, 4.2, 5.3, and 7% for all volume fractions of 20, 25, 30, and 35%, respectively.

### 4.3. Experimental and Numerical Results of Sisal/Gypsum Composites

**Figure 20:**
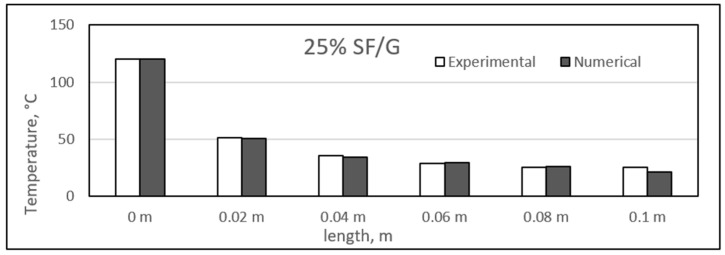
Comparison between experimental and numerical results of 25% sisal/gypsum.

**Figure 21:**
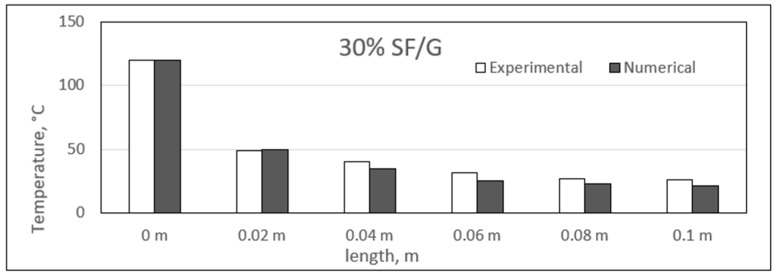
Comparison between experimental and numerical results of 30% sisal/gypsum.

**Figure 22:**
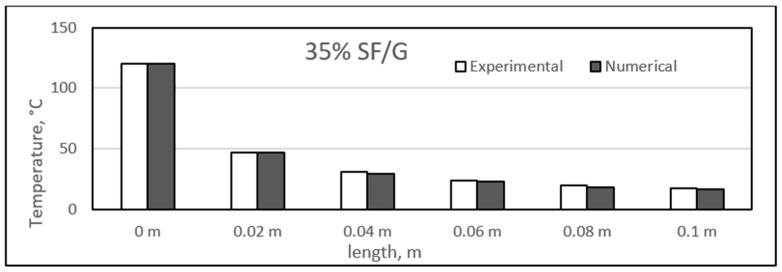
Comparison between experimental and numerical results of 35% sisal/gypsum.

Figure 19, Figure 20, Figure 21 and Figure 22 show a comparison between the experimental and numerical results obtained for heat conduction by using different percentages of sisal fibre in gypsum. The figures show good agreement between both the numerical and experimental results. This provides some reassurance that the ANSYS model is working correctly and is suitable for simulating the heat behaviour of composites. The figures show that there is a slight difference exists between experimental and numerical studies assessment of heat transfer along the composite length. However, it has also been observed that for some composite samples at particular lengths, the difference between the two studies was almost negligible. The 25% composite showed similarity between the two studies at lengths of 0.08 and 0.1 m, and similar results were seen for the 25% composite at these two lengths.

However, the experimental study showed slightly higher heat conduction than theoretical values at different lengths for all composite samples. Even though both studies showed a uniform decrease in the temperature values along the composite lengths, at some point, the experimental temperature was higher. One exception was the point at a length of 0.02 m for the 25% composite, at which the numerical temperature was nearly 3 °C higher than the experimental temperature. When such a case is compared with other samples of sisal mixed with gypsum, the experimental temperature might involve any inaccuracy because of the affection of any operating conditions.

The composites experienced a major temperature drop at the length of 0.02 m, and further, a limited temperature variation existed. An approximate average decrease of 67 °C in the temperature was obtained from 0 to 0.02 m. The temperature values along the length of the composites are inconsistent with the results obtained in previous studies [52]. In the place of sisal fibres, the wheat straw fibres were used to make composites with natural plastic materials. A wide variety of fibre percentages ranging from 0 to 75% were used, and the composites were studied at different temperatures ranging from 10 °C to 40 °C. The composites were found to have similar heat insulation capabilities along their respective lengths [52]. Another similar study was conducted by Chikhi and Agoudjil [28]. On which date, palm fibres were used to form composites with gypsum. It was mentioned that the fibres were less conducive as compared to the gypsum alone, and a considerable drop in temperature was observed with increasing percentage of fibres in the composites.

### 4.4. Experimental and Numerical Results of Glass/Gypsum Composites

**Figure 23:**
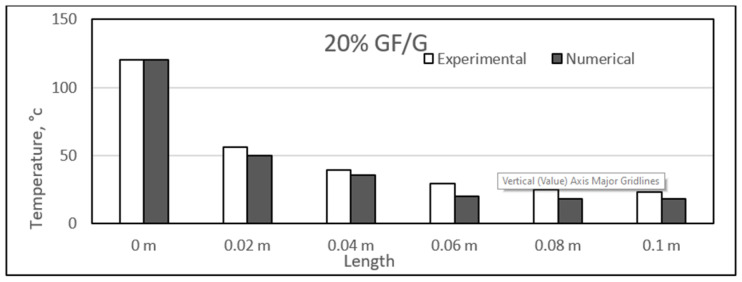
Comparison between experimental and numerical results of 20% glass/gypsum.

**Figure 24:**
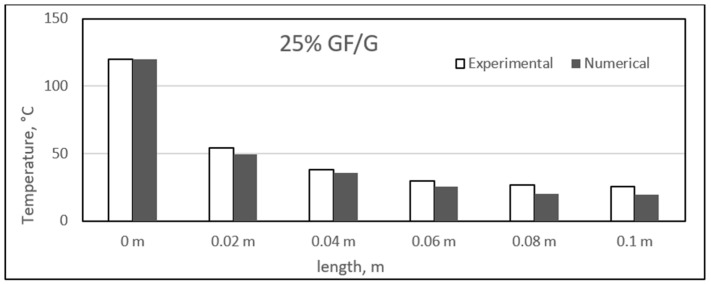
Comparison between experimental and numerical results of 25% glass/gypsum.

**Figure 25:**
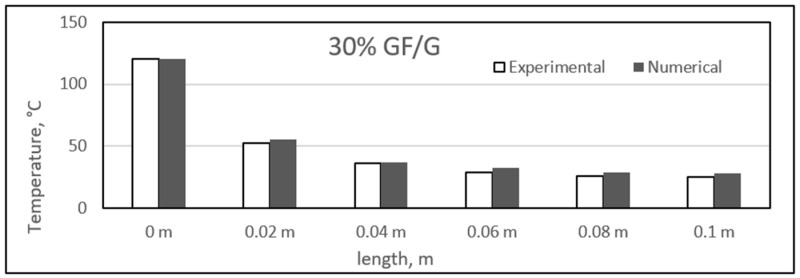
Comparison between experimental and numerical results of 30% glass/gypsum.

**Figure 26:**
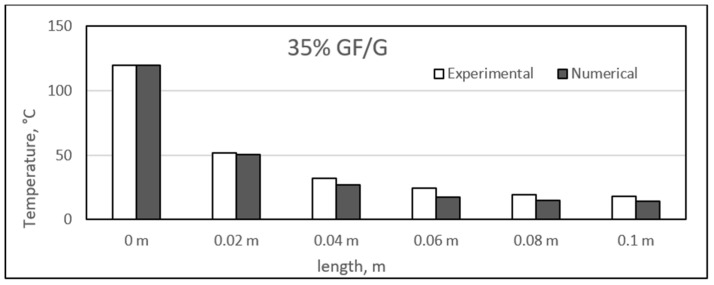
Comparison between experimental and numerical results of 35% glass/gypsum.

Similar to the sisal fibre composites, the difference in the experimental and numerical values was found for the different glass fibre composite samples. The results for the glass fibre composites are shown in the figure, ranging from Figure 23, Figure 24, Figure 25 and Figure 26. In addition, similar results were also found between both experimental and numerical studies, but in this case, two of the samples showed similarity in the results of both studies at particular lengths. However, in contrast to the sisal fibre composites, a number of numerical temperatures values were higher as compared to the experimental temperatures. For example, the 20%, 30%, and 35% glass fibre composites showed such trends.

Overall, a uniform decrease in the results was also obtained along the length of the composite samples. As before, these composites also experienced a major temperature drop at a length of 0.02 m. Further along the length, the temperature variation was very much limited, and a smooth decrease in temperature was observed. The approximate average decrease in temperature from 0 to 0.02 m length was around 68 °C, and further along the length, this decrease ranged from 2 to 9 °C. Recently, a study developed a mathematical model and analysed the thermal conductivity patterns of glass fibre boards. The fibre board samples had different percentages of glass fibres, and it was found that increasing percentages of fibres provide better insulation. However, it was suggested that the percentage should be in a limiting range for obtaining the maximum insulation properties [53,54]. In our case, however, the lowest temperature was 20 °C, and maximum length was achieved for 20 and 35% glass fibre composites. The rest of the two samples were able to achieve 24 °C as the lowest temperature at full length.

Here, the high percentage of error can be found in the middle section of the sample. This can be due to the fact that the middle sections are far from the boundaries. The long-time of running and finding meshing can be used to reduce that amount of error. This point has been given in the recommendations section.

### 4.5. Influence of Volume Fraction of Fibres

**Figure 27:**
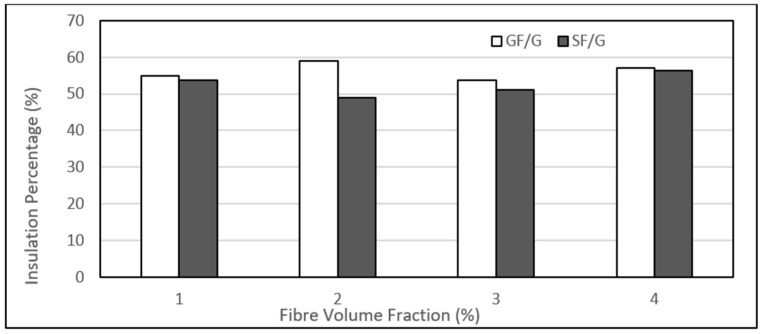
Comparison of the insulation of sisal and glass fibre-gypsum composites with different volume fractions.

A comparison of both sisal and glass fibres in terms of their insulation capabilities in gypsum is shown in Figure 27. A no uniform trend is obtained with increasing fibre volume contents in the gypsum, and a wide difference in the values is obtained for each fibre. The 25% glass fibre composite exhibited the maximum insulation percentage of 59%, and the lowest was 53.6% for 30% glass fibre loading. All glass fibre composites showed higher insulation capability as compared with the sisal fibres composites.

Such a trend was also observed in another study by Patnaik et al. He modelled and experimentally studied the thermal characteristics of glass fibre reinforced epoxy composites having different percentages of glass fibres. He found an increase in the insulation capability of composites with increasing glass fibre contents. However, he found that the insulation capability of the composite was dependent on the glass fibre contents. The insulation capability was increased with the increase in fibre loading, starting from 15%, and, on the other hand, it was decreased slightly beyond 45% loading [55]. Almost similar behaviour is also evident in the figure, and we obtain the maximum insulation percentage at a 25% volume fraction.

## 5. Conclusions

These results have shown that it was possible to construct a theoretical model in ANSYS that produced results that reflected the experimental findings. This analysed used to evaluate the thermal performance of a range of synthetic and natural fibres comparison. First, this evaluation concludes the effect of fibre volume fraction on the composites’ thermal conductivity, which could introduce a new potential to fabricate polymer composites with different volume fractions using sisal fibres and determine the level of porosity in each sample of composites. The second objective was the measurement of heat conductivity of each sample and the development of a mathematical model to correlate the porosity with heat conductivity. A higher volume fraction of natural fibres produced composites with higher insulation performance. Whereas, a higher volume fraction of synthetic fibre produced composites with a lower insulation performance. Overall, the results suggest that the improved performance of natural fibre-based composites was due at least in part to the internal porosity of the fibres. Moreover, this investigation has shown the wide range of thermal performance of synthetic and natural fibre composites. They are affected by many parameters in their manufacture. Moreover, these results highlight the advanced bio-composites and provide a foundation for further research on the molecular basis of resistance heat transfer within the composites.

## Figures and Tables

**Table 1:** Thermal property of composite materials.

Material	Thermal Conductivity (W/mk)	Specific Heat (kcal/kg °C)
Gypsum	0.17	0.26
Sisal fibre	0.07	-
Glass Fibre	0.04	0.16

**Table 2:** Dimensions of the cylinder models and fibres involved.

Volume Fraction %	Dimensional Parameters	Number of Fibres	Radius (cm)
Non	R39	-	2.0
20%	R40	5	0.3111
25%	R51	4	0.3478
30%	R52	4	0.381
35%	R53	3	0.4115

**Table 3:** List of composite samples involved in the model.

Type of Fibre	Samples	Fibre Volume Fraction %
---	Pure gypsum	0%
Glass	GF-Gypsum	20–35%
Sisal	SF-Gypsum	20–35%

**Table 4:** Temperatures differences across glass fibre-gypsum composites of both experimental and numerical results.

Materials	Experimental Results					Numerical Results				
	ΔT1	ΔT2	ΔT3	ΔT4	ΔT5	ΔT1	ΔT2	ΔT3	ΔT4	ΔT5
Pure gypsum	62.7	15.5	12.4	4.0	0.3	63.0	12.5	6.5	2.0	0.5
GF20%G	63.5	17.2	9.8	4.6	1.4	70.1	14.3	15.5	1.6	0.3
GF25%G	65.6	16.4	8.1	3.3	1.2	70.5	14	9.8	5.5	0.5
GF30%G	67.2	16.8	7.1	3.0	0.4	64.2	18.7	4.7	3.4	0.6
GF35%G	68.2	19.7	7.8	4.7	1.2	69.6	23.3	9.76	2.44	0.56

**Table 5:** Errors between the experimental and numerical results of glass fibre-gypsum.

% Errors between Experimental and Numerical Results					
Materials	%Error (ΔT1)	%Error (ΔT2)	%Error (ΔT3)	%Error (ΔT4)	%Error (ΔT5)
PG	3.66	32.3	47.6	50	66
GF20%G	1.1	16.86	58.16	65.2	78.57
GF25%G	6.09	14.63	20.99	66.67	58.33
GF30%G	4.32	11.31	33.8	13.33	50
GF35%G	6.89	18.27	25.13	48.09	53.33

**Table 6:** Temperatures differences across sisal fibre-gypsum composites of both experimental and numerical results.

Materials	Experimental Results					Numerical Results				
	ΔT1	ΔT2	ΔT3	ΔT4	ΔT5	ΔT1	ΔT2	ΔT3	ΔT4	ΔT5
Pure gypsum	62.7	15.5	12.4	4.0	0.3	65.0	20.5	6.5	2.0	0.5
SF20%G	67.4	17.5	7.1	3.3	2.3	68.4	17.4	6.9	3.0	3.2
SF25%G	68.5	15.7	6.8	3.3	0.6	69.2	16.6	4.82	3.4	4.54
SF30%G	71.0	8.8	8.9	4.6	0.6	70.3	14.7	9.7	2.5	1.8
SF35%G	73.0	15.8	7.8	3.9	2.2	72.8	17.7	6.48	4.70	1.63

**Table 7:** Errors between the experimental and numerical results of sisal fibre-gypsum.

% Errors between Experimental and Numerical Results					
Materials	%Error (ΔT1)	%Error (ΔT2)	%Error (ΔT3)	%Error (ΔT4)	%Error (ΔT5)
PG	3.66	32.3	47.6	50	66
SF20%G	1.48	0.57	2.82	9.09	39.1
SF25%G	2.19	6.24	29.12	45.65	65.6
SF30%G	5.2	21.8	8.99	45.65	50
SF35%G	5.75	12.1	16.9	20.5	25.91
